# Current Insights into Molecular Mechanisms and Potential Biomarkers for Treating Radiation-Induced Liver Damage

**DOI:** 10.3390/cells13181560

**Published:** 2024-09-16

**Authors:** Biki Saha, Sneha Pallatt, Antara Banerjee, Abhijit G. Banerjee, Rupak Pathak, Surajit Pathak

**Affiliations:** 1 Medical Biotechnology, Faculty of Allied Health Sciences, Chettinad Academy of Research and Education (CARE), Chettinad Hospital and Research Institute (CHRI), Chennai 603103, India; 2 R&D, Genomic Bio-Medicine Research and Incubation (GBMRI), Durg 491001, Chhattisgarh, India; 3 Division of Radiation Health, Department of Pharmaceutical Sciences, College of Pharmacy, University of Arkansas for Medical Sciences, Little Rock, AR 72205, USA

**Keywords:** ionizing radiation (IR), radiation-induced liver damage (RILD), radiation-induced DNA damage (RIDD), oxidative stress, inflammation, fibrosis, biomarker, miRNA

## Abstract

Highly conformal delivery of radiation therapy (RT) has revolutionized the treatment landscape for primary and metastatic liver cancers, yet concerns persist regarding radiation-induced liver disease (RILD). Despite advancements, RILD remains a major dose-limiting factor due to the potential damage to normal liver tissues by therapeutic radiation. The toxicity to normal liver tissues is associated with a multitude of physiological and pathological consequences. RILD unfolds as multifaceted processes, intricately linking various responses, such as DNA damage, oxidative stress, inflammation, cellular senescence, fibrosis, and immune reactions, through multiple signaling pathways. The DNA damage caused by ionizing radiation (IR) is a major contributor to the pathogenesis of RILD. Moreover, current treatment options for RILD are limited, with no established biomarker for early detection. RILD diagnosis often occurs at advanced stages, highlighting the critical need for early biomarkers to adjust treatment strategies and prevent liver failure. This review provides an outline of the diverse molecular and cellular mechanisms responsible for the development of RILD and points out all of the available biomarkers for early detection with the aim of helping clinicians decide on advance treatment strategies from a single literature recourse.

## 1. Introduction

Liver cancer ranks as the fourth leading cause of death worldwide, with over 800,000 fatalities each year [1,2]. According to Rumgay et al. (2022), the incidence of liver cancer is anticipated to rise by 55.0%, with deaths increasing by 56.4% from 2020 to 2040 [3]. Consequently, primary liver cancer remains a major global health concern. The most common types of primary liver cancer are hepatocellular carcinoma (HCC; accounting for around 90% of primary liver cancer cases) and intrahepatic bile duct cancer (cholangiocarcinoma) [4]. HCC is characterized by complex pathogenesis, high potential for malignancy, and a tendency for metastasis and recurrence. The majority of liver cancer cases are diagnosed in the advanced stages, which severely restricts treatment possibilities.

Recently, various multidisciplinary treatment modalities have been recommended for managing HCC, encompassing procedures like surgical excision, chemotherapy, radiotherapy (RT), and combined therapeutic regimens [5,6]. Among various treatment modalities, RT has emerged as a viable approach to address intermediate-stage HCC and cases of inoperable liver cancer [7,8]. Moreover, recent studies indicate that the use of RT in treating elderly patients suffering from HCC has significantly increased over the years [9]. RT has demonstrated efficacy across all stages of liver cancer and, thus, has become a prevalent treatment modality for combating this particular form of malignancy [10,11].

Primary and secondary liver cancers can be treated effectively by RT because of its number of intrinsic properties. First, RT can effectively eliminate inoperable HCC and cholangiocarcinoma. Secondly, because of the advancements in radiation delivery techniques, a high dose of IR can be delivered precisely in the tumor without damaging surrounding normal liver tissues. Finally, patients with liver metastases are increasingly being recognized as having an oligometastatic disease status that may benefit from targeted liver therapy [12].

In spite of several advancements in RT for treating liver cancer, normal tissue toxicity following RT cannot be completely eliminated. A study indicates that approximately 24.7% of patients experience different degrees of radiation-induced liver disease (RILD) within 22 months after RT [13]. The correlation between RT and RILD was first noticed in 1965 [14]. Although the mechanistic basis of RT-induced RILD is not completely understood, it is believed that radiation-induced highly reactive oxygen species disrupt the structural and functional integrities of key biological entities, such as DNA, lipids, proteins, and their metabolites [15]. Damage to the DNA, specifically double-stranded breaks can lead to cell death, including the death of liver parenchymal (e.g., hepatocytes) and nonparenchymal (e.g., Kupffer cells, sinusoidal endothelial cells, and hepatic lymphocytes) cells, through various mechanisms, such as apoptosis, necrosis, necroptosis, and ferroptosis. Moreover, surviving but damaged parenchymal and nonparenchymal liver cells can lose their genetic or functional integrity, leading to excessive inflammation or the generation of fibrotic molecules, which may contribute to the development of liver fibrosis and cirrhosis at a later stage of life. Therefore, radiation injury to normal liver tissue represents a crucial limiting factor of RT.

Additionally, in the process of generating an allogeneic bone marrow or hematopoietic stem cell transplant, the liver may potentially be exposed to radiation [16,17]. It has long been known that hepatic nonparenchymal cells, such as hepatic stellate cells, sinusoidal endothelial cells, and Kupffer cells, are radiation-sensitive [18]. Following irradiation, these cells release diverse substances (e.g., cytokines, growth factors, and chemokines) that facilitate liver fibrosis, imparting the alteration of liver structure and function [19,20,21]. Radiation-induced hepatic fibrosis poses a highly significant challenge for individuals with RILD [22]. Consequently, investigating the cellular and molecular mechanisms responsible for the pathogenesis of RILD is of the utmost importance for both preventing the progression of RILD and enhancing the effectiveness of RT, ultimately enhancing overall quality of life.

In this review, we present a comprehensive overview and discussion concerning the impact of radiation at the molecular and cellular levels in the context of RILD.

## 2. Clinical Characteristics of RILD: Classic RILD and Nonclassic RILD

The onset of RILD typically occurs within 4–8 weeks after completion of RT, with reported cases ranging from 2 weeks to 7 months after RT [23,24,25]. Approximately 6 to 66% patients undergoing hepatic RT ranging from 30 to 35 Gy develop RILD [26,27,28]. This variability in RILD occurrence can be influenced by factors such as the area of liver in the radiation field, hepatic functional reserve, which refers to the ability of the remnant liver to adequately maintain hepatic function in the postoperative period, including post-radiation therapy and the medical condition of the patients. RILD can be broadly divided into the following two categories: classic RILD and nonclassic RILD. 

Individuals with classic RILD typically manifest prodrome like fatigue, abdominal discomfort, hepatomegaly, enlarged abdominal size, and anicteric ascites within 1–3 months after RT [27,28]. Moreover, alkaline phosphatase levels elevate more than two-fold compared to sham-irradiated control cohort, while transaminase and bilirubin levels remain within standard ranges [28]. Hepatic veno-occlusive disease (VOD), which is characterized by central vein lumina blockage, brought on by erythrocyte entrapment within reticulin and collagen networks, is one of the hallmark features of classic RILD [29,30]. Erythrocyte entrapment leads to vascular congestion, reducing the delivery of oxygen to the central zone, causing centrilobular hepatocyte death and inner hepatic plate atrophy, ultimately resulting in hepatic dysfunction. Additionally, hepatic stellate cells’ (HSCs) activation, contributing to hepatic fibrosis, is a common feature in patients suffering from classic RILD [31].

Nonclassic RILD frequently present with pre-existing chronic liver conditions such as cirrhosis and viral hepatitis, manifesting a higher degree of hepatic dysfunction characterized by jaundice and markedly elevated serum transaminases (exceeding five times the standard levels) rather than alkaline phosphatase levels [26,32]. Compared to noncarrier cohorts, those with an active hepatitis B virus infection are more likely to develop nonclassic RILD. Liver cell loss, liver dysfunction, hepatic sinusoidal endothelial death, and activation of hepatic stellate cells (HSCs) are observed in nonclassic RILD cases. The administration of hepatic RT to nonclassic RILD patients impedes the capacity for hepatocellular regeneration, ultimately leading to irreversible hepatic failure [33,34].

## 3. Pathophysiological Changes in the Liver Due to Radiation Exposure

**Figure 1:**
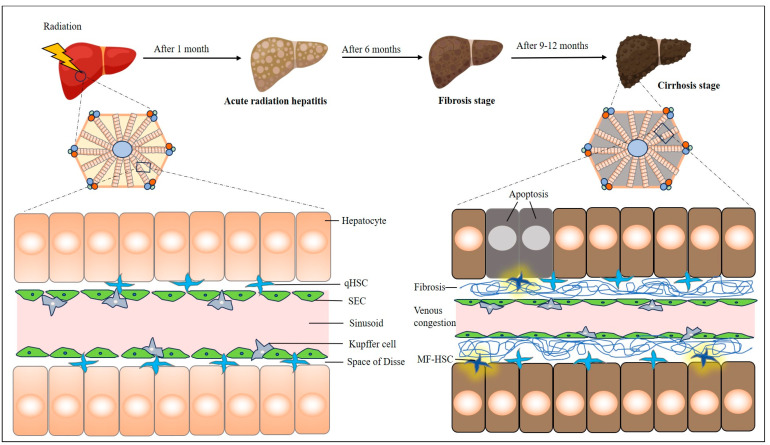
A schematic diagram of pathophysiological changes in the liver due to radiation exposure. MF-HSC—myofibroblastic hepatic stellate cell; qHSC—quiescent hepatic stellate cell; SEC—sinusoidal endothelial cell.

Prior clinical studies demonstrated that patients undergoing hepatic RT are susceptible to venous congestion, which usually occurs within three months [35]. Venous congestion results from the deposition of fibrin and collagen on the inner wall of sinusoids and central veins, leading to an elevation in portal pressure, which is a major contributing factor to RILD. The pathological characteristics of RILD can be divided into the following four stages: acute radiation hepatitis, prehepatic fibrosis, liver fibrosis, and liver cirrhosis. Acute radiation hepatitis typically appears within one-month postirradiation, showing dilation, congestion, and hemorrhage in small blood vessels in the liver. Prehepatic fibrosis usually becomes evident between one and three months after RT. The liver fibrosis stage, often observed around six months postirradiation, is distinguished by hepatic sinusoidal capillarity. Electron microscopy examination reveals that deposition of collagen fibers is at a maximum in the damaged tissues. Liver cirrhosis typically develops 9–12 months after RT, characterized by a noticeable increase in reticulum fibers, laminin, and collagen within the walls of small blood vessels and sinuses (Figure 1).

Exposure to IR can induce both acute and chronic symptoms in the liver. Early radiation effects encompass DNA damage, oxidative stress, and enhanced ROS generation, leading to apoptosis of hepatocytes and triggering severe inflammation, while the delayed effects of radiation on the liver is progressive and irreversible. The clinical signs of RILD pose challenges in detection and have the potential to advance to liver failure [35].

RILD develops by complex multicellular reactions that are associated with the modifications in the vascular architecture, resulting from increased deposits of collagen, production of inflammatory cytokines, and abnormal secretion of growth factors. Under normal physiological conditions, quiescent hepatic stellate cells (qHSCs) are located in the Disse space next to hepatocytes, while sinusoidal endothelial cells (SECs) and Kupffer cells (KCs) are aligned within the sinusoids [36]. After exposure to radiation, SECs undergo apoptosis and, subsequently, release various soluble mediators, including TNF-α, which in turn promotes HC apoptosis and activates KCs. The damaged SECs initiate the coagulation cascade, which results in the deposition of fibrin, triggering thrombosis in the central vein. Further, activated KCs release TGF-β, increasing the susceptibility of hepatocytes to radiation-induced cell apoptosis. This ultimately leads to hepatocyte death, acute liver damage, and liver dysfunction. By interacting with pattern recognition receptors (PRRs), such as nucleotide oligomerization domain (NOD) and nod-like receptors pyrin domain-containing 3 (NLRP3) protein complexes, KCs induce the formation of inflammasomes, which in turn activate T cells and HSCs, as well as damage liver cells. Kupffer cells secrete TGF-β and galectin-3, activating quiescent HSCs through the JNK and p38 MAPK pathways. This activation promotes the proliferation and differentiation of qHSCs into myofibroblastic hepatic stellate cells (MF-HSCs) [35]. When MF-HSCs build up, extracellular matrix protein deposition may increase, which might result in progressive liver fibrosis in patients with RILD [31]. The degree of hepatic fibrosis is strongly correlated with TGF- β level [37]. Following liver damage, apoptotic or balloon-shaped hepatocytes produce Hh (Hedgehog) ligands, which then stimulate the growth of Hh-responsive cells like HSCs and SECs [38,39]. These cells engage with the Hh signaling pathways. Previous studies suggest that the Hh signaling pathway plays a crucial role in the acute, as well as delayed, effects of radiation on the liver, potentially by activating HSCs and SECs [40,41]. Inhibition of the Hh signal may reduce the activation of these cells and, subsequently, alleviate the degree of liver fibrosis caused by radiation.

It is widely acknowledged that TGF-β contributes to the development of RILD. In a preclinical rat model, it has been demonstrated that a dose-dependent increase in TGF-β1 expression, precisely in hepatocytes in the pericentral region of the liver, after 9 months of irradiation [37]. Additionally, a correlation was noted between the percentage of hepatocytes that were positive for TGF-β1 and the extent of fibrosis [42]. Rats who undergo partial liver irradiation show a large increase in TGF-β1 mRNA levels, which can increase by up to 3.6 times compared to sham-irradiated controls in less than a month, even in the absence of severe fibrosis in subsequent biopsies [43].

## 4. Molecular Mechanism of Radiation-Induced Liver Damage

**Figure 2:**
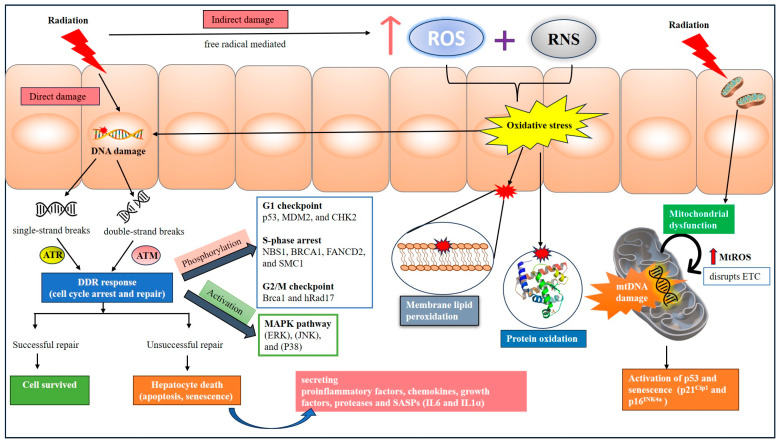
A schematic diagram exhibiting the molecular mechanisms involved in radiation-induced DNA damage. ATM—ataxia telangiectasia mutated; ATR—ataxia telangiectasia and Rad3-related; BRCA1—breast cancer type 1 susceptibility protein; CHK2—checkpoint kinase 2; ERK—extracellular signal-regulated kinase; ETC—electron transport chain; FANCD2—Fanconi anemia group D2 protein; IL1α—Interleukin-1α; IL6—Interleukin-6; JNK—Jun N-terminal kinase; MAPK—mitogen-activated protein kinase; mtROS—mitochondrial reactive oxygen species; NBS1—Nijmegen breakage syndrome protein 1; RNS—reactive nitrogen species; ROS—Reactive oxygen species; SASP—senescence-associated secretory phenotype; SMC1—structural maintenance of chromosomes protein 1.

IR inflicts damage to the biological molecules through both direct and indirect mechanisms. The direct effect entails radiation-induced damage to crucial organic biological macromolecules, like nucleic acids and proteins. Among these, radiation-induced DNA double-strand breaks are highly fatal [44]. DNA double-strand breaks can impair the biological functions of DNA, impacting essential cellular processes [45,46]. Indirect effects are exerted by reactive oxygen species (ROS) and reactive nitrogen species (RNS) generated when radiation ionizes cellular water molecules, which in turn adversely affect biological molecules, including the DNA (Figure 2). ROS such as superoxide (which is highly unstable) can react with nitric oxide to form relatively stable peroxynitrite anions (ONOO−), which is known to cause DNA damage [47]. Thus, radiation-induced DNA damage (RIDD) manifests through a combination of direct physical interactions at the molecular level and the generation of reactive species that initiate chemical alterations in DNA [48,49,50].

### 4.1. RIDD and Cell Senescence

When cells encounter DNA damage, they initiate intricate cellular networks known as DNA damage response (DDR). This response is a vital mechanism in the body’s defense against radiation injury and is crucial in both the onset and progression of RILD.

Cellular senescence following radiation exposure is a form of DDR. Senescence allows cells to detect DNA damage, halt the progression of the cell cycle through checkpoints, and trigger suitable DNA repair mechanisms. This process serves as a protective mechanism to prevent the propagation of genetically compromised cells that could potentially lead to malignancy or other detrimental outcomes [51]. When DNA damage remains unrepaired, cells may undergo senescence [52,53].

Important components of DDR include the DNA-dependent protein kinase catalytic subunit (DNA-PKcs), ataxia telangiectasia and Rad3-related (ATR), and ataxia telangiectasia mutated (ATM). These components are activated based on the type of DNA damage. For example, ATR is activated to repair single-stranded DNA breaks (SSBs), while ATM and DNA-PKcs react mainly to DNA double-strand breaks (DSBs) [54]. Phosphorylation of substrates by these kinases plays crucial roles in orchestrating cell-cycle checkpoints, determining cell fate (including cell death), and facilitating DSB repair [55]. For instance, ATM can undergo activation in response to radiation through processes like intermolecular autophosphorylation and dimer dissociation [56]. Upon activation, ATM kinase phosphorylates a variety of proteins, contributing to different facets of the DNA damage response. In the G1 checkpoint, ATM phosphorylates proteins, including p53, MDM2 (murine double minute 2), and CHK2 (checkpoint kinase 2). These phosphorylation events determine the responses of cells to DNA damage, which may lead to a temporary halt to cell-cycle progression depending on the extent of DNA damage or induction of apoptosis. During transient arrest at the S-phase following radiation exposure, substrates such as NBS1(Nijmegen breakage syndrome protein 1), BRCA1 (breast cancer type 1 susceptibility protein), FANCD2 (Fanconi anemia group D2 protein), and SMC1 (structural maintenance of chromosomes protein 1) are phosphorylated by ATM, triggering cell-cycle arrest to allow time for repair processes to occur. Additionally, in the G2/M checkpoint, ATM targets proteins such as BRCA1 and hRad17 are phosphorylated, contributing to cell-cycle arrest for repair following radiation-induced damage [56] (Figure 2).

Additionally, ATM/ATR phosphorylates the MAPK superfamily in response to IR. This involves the activation of the traditional components of the MAPK pathway, including p38MAPK (P38), c-jun N-terminal kinase (JNK), and extracellular signal-regulated kinase (ERK) [57]. After being exposed to IR, these pathways get activated, which affects biological responses such as apoptosis and cell division. In particular, cell proliferation and apoptosis are promoted by the dose-dependent activation of ERK, ATM, and AKT in response to DSBs induced by IR [58]. Similarly, radiation-induced JNK activation triggers apoptosis in an ATM-dependent manner [59]. To clarify the binding location of JNK substrates and the role of other kinases in this process more research is necessary [60]. Furthermore, radiation-induced DNA damage triggers the activation of the p38 enzyme, which influences cell division by adjusting the degree of oxidative stress [61].

Le et al. (2010) [62] validated that following exposure to a sublethal dose (8 Gy total body irradiation) of radiation in mouse tissues (liver, brain, and lung tissues of C57BL ⁄ 6 mice), liver senescence markers, including P53-binding protein 1 (53BP1) and p16, exhibited significant upregulation shortly after exposure, followed by a gradual decline, with persistence observed for up to 45 weeks. Although damaged cells were selectively eliminated, a notable abundance of senescent markers persisted compared to normal tissue levels. This suggests that while the cellular damage induced by radiation triggers the elimination of some affected cells, a significant portion remains in a senescent state, indicating a sustained impact on liver tissue homeostasis over an extended period.

A recent study [63] demonstrated that a single dose of 25 Gy induced hepatocyte senescence in rats, similar to the effects observed with a hepatectomy of approximately 40% of the liver mass. Various markers of cellular senescence were observed in irradiated hepatocytes, including increased expression of SA-β-gal, augmented cell size, upregulations of p16 and p21, and activation of senescence-associated secretory phenotypes (SASPs), such as IL6 and IL1α. Following senescence, these cells began secreting several proinflammatory cytokines, chemokines, growth factors, and proteases, thereby creating a proinflammatory microenvironment that influences nearby cells either in an autocrine or paracrine manner. These phenotypic changes are collectively referred to as SASPs, indicating a significant alteration in cellular behavior following exposure that contributes to tissue inflammation and potentially impacts neighboring cellular function [64].

The accumulation of a significant number of senescent cells following IR exposure contributes to the disruption of liver regeneration and homeostasis [12]. This disturbance not only results in aberrant liver structure and function but also increases the risk of irreversible liver failure, recurrence of hepatitis B, and even the development of liver cancer. Senescent cells, with their altered secretory profiles and impaired ability to proliferate, can disrupt the normal tissue environment and promote chronic inflammation, thereby exacerbating liver damage and impairing the organ’s capacity to recover from injury. Over time, the persistence of senescent cells can contribute to the progression of liver diseases, including fibrosis, cirrhosis, and, ultimately, hepatocellular carcinoma.

### 4.2. Oxidative Stress

The indirect impact of IR occurs when the energy from radiation does not immediately affect the biological components of cells but rather interacts with the cellular water molecules. This interaction triggers the production of numerous reactive oxygen species (ROS), such as •O_2_–, •OH, and H_2_O_2_ [52,65]. IR can trigger inducible nitric oxide synthase (NOS) to produce nitric oxide (•NO), a type of ROS, through a series of redox reactions. While •NO is largely unreactive with most cellular components, except for heme, and it interacts with superoxide (•O_2_–) to form peroxynitrite (ONOO−) [66]. These free radicals are highly reactive and initiate oxidative reactions in cells within milliseconds of radiation exposure. These highly reactive free radicals attack vital cellular components, leading to cell damage and may cause cell death if not repaired [67]. Radiation exposure to the whole body or liver generates ROS, which can adversely affect various biological components like proteins [68,69], lipids [70,71], and nucleic acids [65,66] in the liver cells, triggering RILD (Figure 2).

In addition, IR-induced post-translational modifications, such as the carbonylation [68] and nitration [69] of liver proteins, enhance cellular oxidative stress, which can contribute to the pathogenesis of RILD. A study demonstrated that carbonylation of liver proteins peaks at 48 h post-IR exposure [68]. Notably, specific proteins, such as carbonic anhydrase 1, enolase, and regucalcin, exhibited heightened levels of carbonylation, suggesting alterations in metabolic processes within the liver [68]. This accumulation of carbonylated proteins can lead to structural and functional impairments, as these extensively oxidized proteins become difficult to degrade. Additionally, nitration of liver protein following irradiation can further exacerbate these detrimental effects on protein function and integrity [72]. 

IR-induced lipid peroxidation involves the oxidative transformation of unsaturated fatty acids into various metabolites, such as malondialdehyde (MDA) and lipid peroxides [73]. Within liver parenchymal cells, radiation-induced ROS are primarily generated within mitochondria, microsomes, and peroxisomes. IR-induced excess ROS generation can trigger peroxidation of unsaturated fatty acids within liver cell membranes, giving rise to lipid peroxides. The process of membrane lipid peroxidation has multifaceted effects on cellular function. For example, it can increase the rigidity of membranes, impacting their flexibility and cellular absorption processes. This alteration in membrane properties of hepatocytes can subsequently lead to decreased activity of membrane-bound enzymes, impairing their function and affecting vital cellular processes. Additionally, membrane lipid peroxidation can disrupt membrane receptor activity, potentially interfering with cell signaling pathways and cell-to-cell communication. Furthermore, it can alter membrane permeability, influencing the passage of molecules and ions across the membrane and disrupting cellular homeostasis [52]. All of these changes in the membrane of hepatocytes contribute to the pathogenesis of RILD.

### 4.3. miRNAs in RILD

IR can also alter the expression levels of different microRNAs (miRNA) [74,75]. miRNAs represent a category of diminutive noncoding RNAs that play a role in the posttranscriptional control of genetic information. These molecules exert significant influence over a multitude of cellular processes, such as cellular proliferation, specialization, maturation, and programmed cell death [76]. Through their effects on cell-cycle checkpoints, apoptosis, DNA repair and damage response (DDR) gene expression, and the tumor microenvironment (TME), miRNAs may influence the radiosensitivity of hepatocytes. Many miRNAs induce double-strand breaks (DSBs) in an ATM-dependent way in response to DDR [77]. Martin et al. showed that IR promotes DSB formation by suppressing miR-335 expression through an ATM-dependent pathway [78]. Furthermore, ATM-mediated phosphorylation of p53 results in the release of p53 from its inhibitor MDM-2, thereby activating p53. This activation triggers the expression of several miRNAs, including miR-34a and miR-192 [77,79,80].

IR-induced miR-34a contribute to liver damage by triggering cytokine release, DNA damage, antioxidant molecules, cellular senescence, and cell-cycle inhibition [81]. miR-34a modulate cellular apoptosis by modulating levels of phosphorylated key proteins within the MAPK signaling pathway via the expressions of MAP3K9 and MAP3K10 [82,83]. After IR exposure, elevated levels of miR-34a-5p directly reduced the synthesis of a protein known as Krüppel-like factor 4 (KLF4) and caused liver cell death [83]. Bcl-2, the downstream target of miR-34a, has been shown to regulate radiosensitivity [84]. Several cell types exhibit increased radiosensitivity due to the overexpression of miR-34a and downregulation of Bcl-2 expression [85]. IR causes downregulation of miR-495. Elevation of miR-495 levels could potentially alleviate RILD by modulating the interaction between the transcription factor 1 (Sp1) and endothelial nitric oxide synthase (eNOS) pathways. 

Radiation contributes to extracellular vesicle (EV) release in a dose-dependent manner [86,87]. Interestingly, miRNAs can also be passed from irradiated cells to nearby cells through EVs, exacerbating RILD [88]. EVs of irradiation cells contain miR-21 and miR-34c, which might cause the bystander effect in nonirradiated cells [89,90]. miR-146a-5p plays a crucial role in regulating lipopolysaccharide (LPS)/TLR4 signaling. Increased levels of miR-146a5p have been found to reduce liver cell death and scarring, inhibiting cellular growth, release of inflammatory molecules, and cellular activation following irradiation in a TLR4-dependent manner [87]. Although recent advancements have improved our understanding of miRNA production and regulation during DDR after radiation exposure, many uncertainties still exist. These unresolved issues pose challenges for managing RILD in clinical practice. 

### 4.4. Mitochondrial DNA Damage

Mitochondria regulate cellular ROS generation. The liver is highly rich in mitochondria because of its high metabolic activity, and it has been found that under normal physiological conditions, the liver accounts for approximately 20% of the total oxygen consumption by the body. Radiation adversely affects mitochondrial function. A study showed that exposure to 30 Gy can induce hepatic mitochondrial damage by enhancing ROS generation [91].

IR changes the permeability of the mitochondrial membrane, which impairs the electron transfer chain and oxidative phosphorylation, leading to an accumulation of superoxide beyond the capacity of superoxide dismutase (SOD) and catalase (CAT) to neutralize in a timely manner. The excess superoxide triggers endogenous ROS generation [92,93]. IR also prompts the hydrolysis of cellular components, resulting in the formation of hydroxyl radicals (•OH), causing a notable surge in exogenous ROS levels. This cascade eventually leads to cellular senescence or death because of mitochondrial malfunction [92].

Radiation causes damage to mitochondrial DNA (mtDNA) compared to nuclear DNA, as it lacks histones and an efficient DNA repair system. Consequently, mitochondria with damaged DNA may transmit to progeny cells, leading to prolonged harm and even secondary cancer development [63]. MtDNA exclusively composed of coding DNA and mutations directly impact proteins involved in the mitochondrial electron transport chain (ETC), leading to mitochondrial dysfunction. In a study, a small animal image-guided radiation treatment platform was used to deliver a single dosage of 50 Gy of radiation to focus sites in mouse livers. The sequencing results revealed a notable rise in frameshift mutations in two of the ETC complex IV genes, one of the ETC complex III genes, and seven of the ETC complex I genes. Additionally, their results imply that radiation can cause mtDNA mutations, which in turn activate p53 and senescence-related genes, including as p21^Cip1^ and p16^INK4a^ [94] (Figure 2).

Mitochondrial deacetylase sirtuin 3 (SIRT3), the primary protein deacetylase within mitochondria, plays a pivotal role in regulating the mitochondrial response to IR and other oxidative stresses. Being exclusively localized in mitochondria, SIRT3 governs key mitochondrial processes such as protein deacetylation. Through its impact on the hydrogen peroxide cascade and hydrogen peroxide signaling pathway, SIRT3 has been shown to be important in regulating the pathogenesis of RILD in mice [70,95]. Through their interactions with SIRT3, the superoxide anion radical (•O_2_–) and FOXO3a also have an effect on RILD [70,96,97]. 

SIRT3 serves a vital role as a stress response protein by regulating levels of ROS through modifying antioxidant enzymes like MnSOD, enhancing their activity. SIRT3 affects Mn-SOD’s function by controlling its deacetylation [98]. IR triggers deacetylation of Mn-SOD in murine liver, boosting its activity. This suggests that the SIRT3/Mn-SOD axis is activated by IR and could counteract oxidative stress and injury [99]. Nonetheless, the precise mechanism by which SIRT3 influences RILD through Mn-SOD regulation remains unclear. The Sirt3^−/−^ mice showed greater levels of DNA damage, protein oxidation (3-nitrotyrosine), immune cell infiltration, and bile duct damage six months after 24 Gy exposure to liver as compared to controls. Furthermore, Sirt3^−/−^ mice showed upregulation of fibrotic markers (procollagen 1 and α-SMA) and proinflammatory chemokines (IL-6, IL-1β, and TGF-β) following irradiation. Long-term liver damage brought on by radiation in Sirt3^−/−^ mice may also be mediated via hydrogen peroxide and hydroperoxide-sensitive signaling pathways, depending on alterations in the enzymatic activity of catalase, glutathione reductase, and hydrogen peroxide. It is interesting to note that neither Sirt3^−/−^ nor Sirt3^+/+^ mice’s MnSOD activities differed significantly between the irradiation and sham groups [95].

### 4.5. Inflammation and Fibrosis

Radiation can cause cell death by inducing mitotic catastrophe, apoptosis, necrosis, necroptosis (or secondary necrosis), autophagy, and senescence. From an immunological standpoint, radiation-induced cell death can be divided into the following two categories: immunogenic and nonimmunogenic cell death. Necrosis and necroptosis trigger an immune response, making them immunogenic, while apoptosis is anti-immunogenic [100]. The body’s reactions to radiation can provoke inflammation, resulting in the release of different soluble mediators and changes in immune cell surface receptors. These responses are known to be crucial in the development of radiation-induced toxicities in normal tissues.

**Figure 3:**
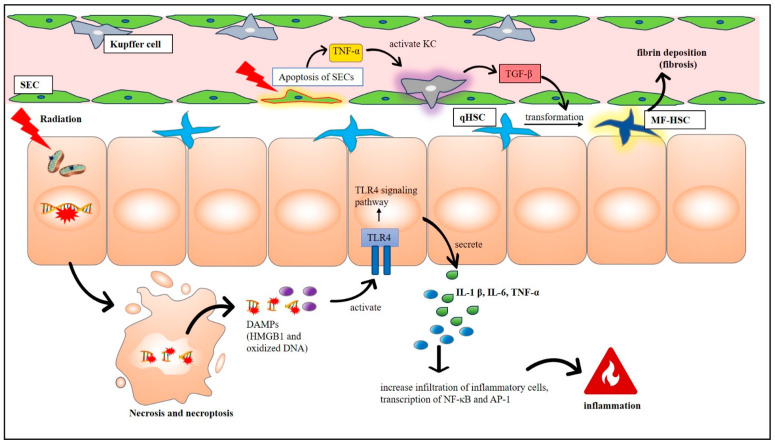
A schematic diagram of radiation-induced inflammation and fibrotic changes contributing to liver damage. AP-1—activator protein-1; DAMP—damage-associated molecular pattern; HMGB1—High-mobility group box 1; IL-1β—interleukin-1beta; IL6—interleukin-6; KC—Kupffer cell; MF-HSC—myofibroblastic hepatic stellate cell; NF-Κb—Nuclear factor kappa B; qHSC—quiescent hepatic stellate cell; SEC—sinusoidal endothelial cell; TGF-β—transforming growth factor beta; TLR4—toll-like receptor 4; TNF-α—tumor necrosis factor alpha.

Cell death because of IR-induced mitochondrial or DNA damage can activate numerous signaling pathways that prompt immune cells to react. Damaged cells may release many inflammatory substances, such as cytokines and chemokines, which may cause persistent damage to various organs, including the liver. Zhou et al. (2012) reported that IR-induced death of liver nonparenchymal cells, such as KCs, SECs, and HSCs, releases a variety of cytokines [101]. Furthermore, exposure to radiation can cause the liver to produce TNF-α, IL-6, and IL-1β. Liver cells exposed to radiation and cultured in a TNF-α-rich medium produced by irradiated liver macrophages experience heightened apoptosis compared to the irradiated liver cells cultured in normal cell culture medium [19]. However, Chen et al. (2015) revealed that radiation primarily induces apoptosis in sinusoidal endothelial cells (SECs) rather than hepatocytes [102]. Injury to SECs can disturb microcirculation, triggering leukocyte activation and ultimately causing damage to hepatocytes [20,102]. Activated KCs can release TGF-β, which is a crucial factor in liver fibrosis, particularly in regulating ECM proteins in RILD (Figure 3). Early-stage IR in SD rats show varying expressions of TGF-β1 and TGF-β3 mRNAs, with TGF-β1 favoring fibrosis promotion [43]. Rats in RILF (radiation-induced liver fibrosis) models with anti-transforming growth factor-β (TGF-β) intervention exhibit reduced oxidative stress damage and notably improved liver fibrosis, underscoring TGF-β’s significance in RILD [103,104,105].

Damaged mitochondrial DNA (mtDNA) contributes to immune activation. Oxidized mtDNA may be released into the cytosol after mitochondrial death and attach to the NLRP3 inflammasome, thereby activating it [106]. Furthermore, radiation exposure can raise the concentrations of certain inflammatory cytokines, which can trigger the activation of the apoptosome and inflammasome during the process of apoptosis [107,108,109]. The maturation and production of inflammatory cytokines, including IL-1β and IL-18, are facilitated by the activation of the inflammasome [110]. On the other hand, radiation exposure causes reactive oxygen species (ROS) to be produced, which in turn causes inflammasome activation [111,112,113].

Damage-associated molecular patterns (DAMPs) such as HMGB1 (high-mobility group box 1) and oxidized DNA are released when there is extensive damage to DNA. By connecting DNA damage and immunological responses, these DAMPs cause inflammation when they interact with the immune system via certain ligands [114]. These DAMPs are recognized by pattern recognition receptors (PRRs) called toll-like receptors (TLRs). Radiation exposure has been shown to activate the TLR4 signaling system and increase the expression of TLR4 in liver cells [87,115]. This activation causes the release of cytokines and inflammatory substances, such TNF-α, IL-1, and IL-6 [116]. These factors then promote the infiltration of inflammatory cells and trigger the transcription of activator proteins (AP-1) and NF-κB [117,118]. Liver inflammation and damage are the ultimate outcomes of this procedure.

NF-κB functions as a key element in the response to perilous signals emanating from necrotic or necroptotic cells. The recognition of damage-associated molecular patterns (DAMPs) by TLRs can induce NF-κB signaling through pathways that encompass mitogen-activated protein kinases (MAPKs), DNA-dependent protein kinases (DNA-PKs), and phosphoinositide 3-kinase (PI3K) [119]. NF-κB governs the transcription of diverse growth factors, inhibitors of apoptosis, and molecules crucial for inflammation, such as proinflammatory cytokines, chemokines, inducible nitric oxide synthase (iNOS), cyclooxygenase-2 (COX-2), and vascular adhesion molecules that are essential for the recruitment of leukocytes.

In RILD, proinflammatory cytokines and ROS may trigger the NF-κB signaling pathway [120,121]. Inflammatory injury is made worse by the release of TNF-α and IL-6, which are brought on by NF-κB activation [120]. In hepatocytes and nonparenchymal cells (NPCs) [121], radiation exposure enhances the expression of NF-κB-related genes (e.g., TRAF6, NIK, RELB, IKK, and RELA) implicated in both canonical and noncanonical NF-κB pathways [122]. Furthermore, it has been noted that RILD exhibits increased RelA (p65) expression and nuclear translocation [71,123,124]. Anti-inflammatory and antioxidant drugs can slow the progression of RILD by lowering NF-κB expression, according to several studies [71,123].

The long-term development of radiation-induced fibrosis is partly attributed to immune responses that occur after exposure to IR. Irradiated tissue exhibits the upregulation of several factors, including chemokines, microRNA-21, pro-inflammatory cytokines (like TNFα, IL1, IL-4, IL6, and IL-13), fibrogenic cytokines (TGF-β), chemokines, vascular endothelial growth factor (VEGF), elements of the renin–angiotensin system, and platelet-derived growth factor (PDGF). Fibrosis is caused by these substances, which also stimulate the growth of fibroblasts and the production of ECM metalloproteinases and protein matrix [125,126]. 

Following radiation exposure, TGF-β1 plays a crucial role in normal tissue remodeling by inducing fibrosis via pathways, such as TGF-β1Rho/ROCK and TGF-β1-smad2/3 [127,128]. Collagen and fibronectin build up in the ECM (extracellular matrix) as a result of its inhibition of matrix proteases and enhancement of matrix protein production [129,130]. 

## 5. Biomarkers of RILD

Recognizing RILD poses a significant challenge because of the delayed onset of clinical manifestations, which typically emerge at more advanced stages of the condition. The lack of effective treatment options and the absence of established early biomarkers further complicate the management of RILD. The majority of insights into RILD have been derived from autopsy findings in the late stages of the disease, highlighting the critical need for the identification of early biomarkers. Such biomarkers would be invaluable in guiding treatment adjustments and potentially preventing the progression of liver failure [131]. In this context, the development of both serum and imaging biomarkers is of paramount importance (Table 1).

The evaluation of patients with liver cancer or other malignancies for which radiation therapy may be employed heavily relies on assessing serum biomarkers indicative of liver function. Key measures include serum albumin, bilirubin, and prothrombin time, which are used to evaluate hepatic synthetic function [132]. Additionally, other serum markers, such as ADAMTS13 (a protease involved in cleaving von Willebrand factor), hyaluronic acid levels, and plasminogen activation inhibitor, have been investigated for their potential to predict the risk of veno-occlusive disease (VOD) in patients undergoing bone marrow transplants. However, their predictive value for radiation-induced liver disease (RILD) has not been established [133]. In a Phase II clinical trial involving adaptive stereotactic body radiation therapy (SBRT), variations in serum cytokine levels were observed, where increased hepatocyte growth factor levels and decreased CD40 ligand levels were suggested as potential early indicators of RILD following SBRT [134].

Furthermore, specific microRNAs, including miR-122-3p, miR-141-3p, miR-200b-3p, miR-375, miR-217, and miR-125a-5p, have been identified as potential predictors of liver toxicity [135]. Ng, Sylvia S. W. et al. (2020) [136] explored metabolomic profiles that could serve as early biomarkers for RILD. Following whole-liver irradiation at doses ranging from 10 to 50 Gy, increased levels of certain metabolites related to glucose, amino acid, and nucleotide metabolism were observed in the liver and plasma of mice, with the quantities varying according to the radiation dose. 

Fibrinogen-like 1 (FGL1), also known as hepassocin or hepatocyte-derived fibrinogen-related protein 1 (HFREP1), is a protein produced by hepatocytes and was first identified as being highly expressed in human hepatocellular carcinoma (HCC) [137]. The structure of FGL1 includes a fibrinogen-related domain in its C-terminal region but lacks three functional domains critical for platelet binding, crosslinking, and thrombin sensitivity [138]. J. Chen et al. (2024) [139] showed that FGL1 plays a significant role in the progression of nonalcoholic liver disease. Han et al. (2019) [140] explored the expression of FGL1 in the liver and plasma after radiation exposure. Researchers observed that 30 Gy of liver irradiation caused cell death through apoptosis, necrosis, and autophagy, leading to fibrotic liver changes in mice during the acute and subacute phases. The pattern of FGL1 expression in the liver was linked to liver damage, indicated by injury-related proteins and oxidative stress markers. In human hepatocytes, FGL1 expression confirmed its association with radiation-induced liver injury. The study further measured FGL1 levels in liver tissue and plasma of mice following total body irradiation (TBI) or liver irradiation. FGL1 was most elevated in the liver compared to other organs after TBI, and plasma FGL1 levels correlated well with liver irradiation dose. The findings suggest that plasma FGL1 could be a potential biomarker for detecting acute and subacute radiation-induced liver injury [140].

Noninvasive imaging techniques offer the potential to provide more detailed insights into liver function before therapy, the diverse roles of the liver, and its changes throughout radiation treatment. For example, sulfur colloid single-photon emission tomography (SPECT) uptake has been found to correlate with indocyanine green retention at 15 min (ICG-R15) and has been utilized to monitor liver function [141]. Moreover, studies have demonstrated associations between this imaging technique and overall survival, as well as clinical liver function, in patients undergoing stereotactic body radiation therapy (SBRT) planning [142,143]. The reduction in portal perfusion caused by radiation-induced veno-occlusive disease (VOD) suggests that RILD can potentially be predicted through noninvasive hepatic perfusion measurements. Additionally, a strong correlation has been observed between ICG clearance and residual liver function, as assessed by dynamic contrast-enhanced CT scans and magnetic resonance imaging (MRI), which measure spatially distributed portal vein perfusion across the liver parenchyma following radiation therapy [144,145].

**Table 1:** List of biomarkers employed for RILD detection.

Biomarkers of RILD	Impact	Dose	References
Serum biomarkers			
Experimental study			
Fibrinogen-like 1 (FGL-1)	Correlates with hepatocellular damage after radiation exposure in human primary hepatocytes	0–30 Gy	[140]
MicroRNAs	Upregulations of miR-122 9, miR-34a, miR-192, miR-146a-5p, and miR-495 are downregulated after radiation exposure. IR promotes DSB formation by suppressing miR-335 expression. miR-21 and miR-34c induce the bystander effect in nonirradiated cells	2, 4, and 10 Gy	[146,147]
Metabolomics	Patients with RILD have higher amounts of certain precursors or intermediates in their glucose, amino acid, and nucleotide metabolisms	50 Gy	[146]
Clinical study			
Hepatocyte growth factor (HGF)	Increased in RILD patients	55 Gy	[148]
CD40 ligand	Low levels of CD40L predict toxicity	23–55 Gy	[148,149]
Indocyanine green clearance	Presented as an indicator for liver health	30 Gy	[149]
Hepatic synthetic function test	Extensively utilized technique to forecast RILD and liver function	-	[132]
Imaging biomarkers			
Clinical study			
Sulphur colloid SPECT-CT	Correlates with clinical liver function	37.5–40 Gy	[150]
Dynamic contrast-enhanced CT	Portal vein perfusion and ICG measurements of liver function are correlated	40 Gy	[144]
Portal venous perfusion data	Ability to develop global and local models of liver function for potential radiation treatment adaption	45–85 Gy	[151]

## 6. Conclusions

In conclusion, we summarize the various molecular and cellular pathways that contribute to the pathogenesis of RILD, which may occur during or after abdominal or hepatic RT. Normal tissue toxicity (including liver) from therapeutic radiation constitutes a significant constraint in clinical settings. Radiation damage to the liver can lead to radiation-induced liver fibrosis (RILF) and liver cirrhosis, thereby profoundly influencing subsequent treatments, prognosis, and patient quality of life. Through a complex cascade of interactions, RILD tightly links diverse responses as it unfolds as a multistage, multistep dynamic process. Within this paradigm, a variety of signaling pathways regulate the complex interactions between DNA damage, inflammation, oxidative damage, senescence of cells, fibrosis, and immune system responses.

RILD primarily results from direct DNA damage caused by radiation, which may lead to cellular senescence or cell death depending on the extent and quality of the DNA damage. Moreover, irradiation, which does not cause cell death, may disrupt the homeostasis between oxidants and antioxidants machinery in different types of hepatic cells, leading to enhanced oxidative stress, which may adversely affect the structure and function of proteins, lipids, and nucleic acids, resulting in dysregulated metabolism. On the other hand, a high dose of radiation may cause necrosis and apoptosis of hepatocytes, contributing to inflammatory response in the liver through various pathways. The interplay between damaged hepatocytes and liver nonparenchymal cells further exacerbates inflammation and promotes liver fibrosis.

Mitigating liver tissue damage, re-establishing cellular homeostasis, suppressing inflammation, and reducing cytotoxicity following irradiation are crucial strategies to reduce the risk of developing RILD treatment. Numerous probable therapeutic targets, including RNAs, antioxidants, and immunosuppressants, have been identified. These targets are intended to prevent or mitigate radiation toxicity and suppress inflammation and oxidative stress. However, the clinical success of these therapeutic targets remains to be established. Interestingly, no FDA-approved drugs are available to treat RILD. Moving forward, deeper comprehension of RILD’s molecular and cellular mechanisms is imperative, along with the discovery of additional biomarkers to advance targeted RT, enhance patient quality of life, and reduce toxicity.

## Data Availability

No new data were created or analyzed in this study. Data sharing is not applicable to this article.

## Abbreviations

NO	Nitric oxide
•OH	Hydroxyl radicals
4-HNE	4-hydroxynonenal
53BP1	P53-binding protein 1
8-OHdG	8-hydroxydeoxyguanosine
AP-1	Activator protein-1
ATM	Ataxia telangiectasia mutated
ATR	Ataxia telangiectasia and Rad3-related
BRCA1	Breast cancer type 1 susceptibility protein
CAT	Catalase
CHK2	Checkpoint kinase 2
COX-2	Cyclooxygenase-2
CT	Computed tomography
CYP450	Cytochrome P450
DAMP	Damage-associated molecular Pattern
DDR	DNA damage response
DNA-PKcs	DNA-dependent protein kinase catalytic subunit
DNA-PKs	DNA-dependent protein kinases
DSB	Double-strand break
ECM	Extracellular matrix
ERK	Extracellular signal-regulated kinase
ETC	Electron transport chain
FANCD2	Fanconi anemia group D2 protein
FGL1	Fibrinogen-like 1
FRAP	Ferric-reducing antioxidant power
GSHpx	Glutathione peroxidase
HCC	Hepatocellular carcinoma
HFREP1	Hepatocyte-derived fibrinogen-related protein 1
Hh	Hedgehog
HMGB1	High-mobility group box 1
HSC	Hepatic stellate cell
ICG	Indocyanine green
IL-1B	Interleukin-1beta
IL1α	Interleukin-1α
IL6	Interleukin-6
iNOS	Inducible nitric oxide synthase
IR	Ionizing radiation
JNK	c-Jun N-terminal kinase
KC	Kupffer cell
KLF4	Krüppel-like factor 4
MAPK	Mitogen-activated protein kinase
MDA	Malondialdehyde
MDM2	Murine double minute 2
MF-HSCs	Myofibroblastic hepatic stellate cells
MRI	Magnetic resonance imaging
mtDNA	Mitochondrial DNA
mtROS	Mitochondrial reactive oxygen species
NBS1	Nijmegen breakage syndrome protein 1
NF-Κb	Nuclear factor kappa B
NLRP3	Nod-like receptors pyrin domain-containing 3
NOD	Nucleotide oligomerization domain
NOS	Nitric oxide synthase
NPCs	Non parenchymal cells
NTCP	Normal tissue complication probability.
•O_2_–	Superoxide
ONOO−	Peroxynitrite anion
PDGF	Platelet-derived growth factor
PI3K	Phosphoinositide 3-kinase
PRR	Pattern recognition receptor
qHSC	Quiescent hepatic stellate cell
RIDD	Radiation-induced DNA damage
RILD	Radiation-induced liver disease
RILF	Radiation-induced liver fibrosis
RNS-	Reactive nitrogen species
ROS	Reactive oxygen species
RT	Radiation therapy
SASP	Senescence-associated secretory phenotype
SA-βgal	Senescence-associated β-galactosidase
SBRT	Stereotactic body radiotherapy
SEC	Sinusoidal endothelial cell
SIRT3	Sirtuin 3
SMC1	Structural maintenance of chromosomes protein 1
SOD	Superoxide dismutase
SSB	Single-stranded DNA break
TBARS	Thiobarbituric acid reactive substances
TGF-β	Transforming growth factor beta
TLR	Toll-like receptor
TNF-α	Tumor necrosis factor alpha
VEGF	Vascular endothelial growth factor
VOD	Veno-occlusive disease
αSMA	Alpha smooth muscle Actin
